# Altered Sphingolipid Metabolism in Patients with Metastatic Pancreatic Cancer

**DOI:** 10.3390/biom3030435

**Published:** 2013-07-25

**Authors:** Yixing Jiang, Nicole A. DiVittore, Megan M Young, Zhiliang Jia, Keping Xie, Timothy M. Ritty, Mark Kester, Todd E. Fox

**Affiliations:** 1 Pennsylvania state Hershey cancer institute, Hershey, PA17033, USA; E-Mails: yjiang@umd.edu (Y.J.); nad117@psu.edu (N.A.D.); mkester@hmc.psu.edu (M.K.); 2 Department of pharmacology, Hershey, PA17033, USA; E-Mail: myoung3@hmc.psu.edu (M.M.Y.); 3 Department of gastrointestinal medical oncology, the University of Texas MD Anderson cancer center, Houston, TX77030, USA; E-Mails: zjia@mdanderson.org (Z.J.); kepxie@mdanderson.org (K.X.); 4 Department of orthopedics Pennsylvania state college of medicine, 500 University Drive, Hershey, PA 17033, USA; E-Mail: tmritty@gmail.com

**Keywords:** sphingolipids, pancreatic cancer, ceramide, lipidomics, CXCL10

## Abstract

Although numerous genetic mutations and amplifications have been identified in pancreatic cancer, much of the molecular pathogenesis of the disease remains undefined. While proteomic and transcriptomic analyses have been utilized to probe and characterize pancreatic tumors, lipidomic analyses have not been applied to identify perturbations in pancreatic cancer patient samples. Thus, we utilized a mass spectrometry-based lipidomic approach, focused towards the sphingolipid class of lipids, to quantify changes in human pancreatic cancer tumor and plasma specimens. Subgroup analysis revealed that patients with positive lymph node metastasis have a markedly higher level of ceramide species (C16:0 and C24:1) in their tumor specimens compared to pancreatic cancer patients without nodal disease or to patients with pancreatitis. Also of interest, ceramide metabolites, including phosphorylated (sphingosine- and sphinganine-1-phosphate) and glycosylated (cerebroside) species were elevated in the plasma, but not the pancreas, of pancreatic cancer patients with nodal disease. Analysis of plasma level of cytokine and growth factors revealed that IL-6, IL-8, CCL11 (eotaxin), EGF and IP10 (interferon inducible protein 10, CXCL10) were elevated in patients with positive lymph nodes metastasis, but that only IP10 and EGF directly correlated with several sphingolipid changes. Taken together, these data indicate that sphingolipid metabolism is altered in human pancreatic cancer and associated with advanced disease. Assessing plasma and/or tissue sphingolipids could potentially risk stratify patients in the clinical setting.

## 1. Introduction

Pancreatic cancer is one of the leading causes of cancer related deaths with a five-year survival rate of approximately 5% [1]. While several biomarkers and altered biochemical/metabolite pathways have been putatively identified via proteomics or transcriptomics [2,3], the lipidome has not been rigorously analyzed.

Sphingolipids manifest diverse biological, biochemical and biophysical functions that mediate, in part, cellular proliferation, migration, tumorigenesis apoptosis and inflammation [4,5]. Multiple reviews have implicated dysfunctional sphingolipid metabolism and/or signaling in solid and non-solid tumor models [6,7,8]. Based upon multiple studies denoting alterations in ceramide metabolism or signaling after anti-neoplastic chemo- [4,9,10,11] or radiation- [12] therapy, targeting sphingolipid pathway has become an emerging arena for cancer therapy. A myriad of molecular and pharmacological studies have validated the role of ceramide species and metabolites to regulate tumor growth, migration, or metastasis. However, these studies have largely focused on *in vitro* cell lines and xenograft/syngeneic mouse models. The role of, and regulation of, altered sphingolipid metabolism in cancer development, progression and metastasis remains largely undefined in human patient populations. To address this deficiency, a mass spectrometry-based targeted analysis of sphingolipids in human pancreatic cancer clinical specimens was initiated. 

## 2. Results and Discussion

### 2.1. Deregulation of Sphingolipid Metabolites in Patients with Pancreatic Cancer

**Figure 1:**
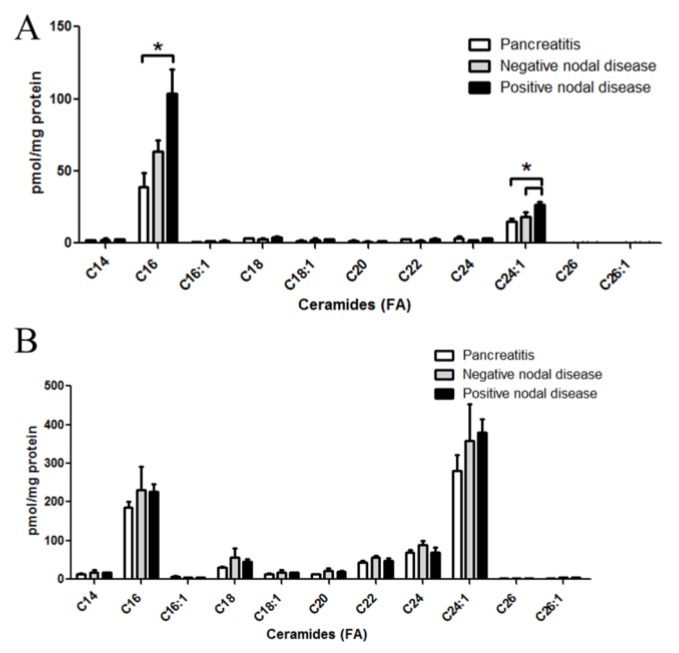
Altered ceramide levels in patient specimens with pancreatic cancer. LC-MS/MS was utilized to quantify ceramides from patient specimens. Ceramides with a d18:1 backbone with fatty acids from 14 to 26 carbons were assessed. Molecular species of ceramides were quantified from pancreas tissues (**A**); and corresponding plasma samples (**B**) from patients with pancreatitis, non-metastatic (nodal negative) pancreatic cancer, or metastatic (nodal positive) pancreatic cancer. * = *p <* 0.05 assessed by *t*-test analysis.

MS-based lipidomic analysis of ceramides from pancreatic tissues revealed discrete and significant changes in specific ceramide molecular species in metastatic pancreatic cancers as compared to either pancreatic cancers without metastases or non-cancerous pancreatitis specimens (Figure 1A). Specifically, palmitate (C16:0)- and nervonic acid (C24:1)- containing ceramides were the most prevalent species detected in all tumor samples. Interestingly, higher tissue levels of C16:0-ceramide (*p* < 0.05) and C24:1-ceramide (*p* < 0.005) were associated with positive regional lymph node metastasis, whereas patients negative for metastasis did not show any significant changes (Figure 1A). Changes in ceramide species were not observed in corresponding plasma samples from any of these patient populations (Figure 1B). 

**Figure 2:**
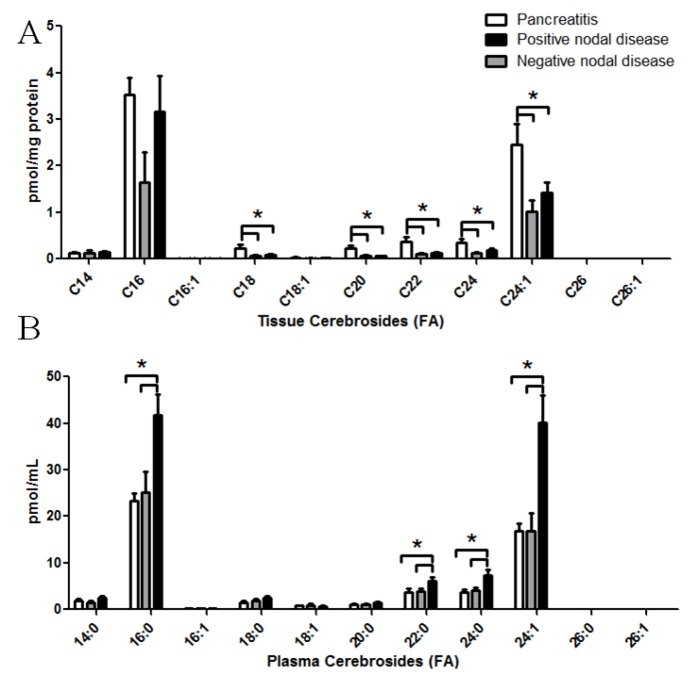
Altered cerebroside levels in patient specimens with pancreatic cancer. LC-MS/MS was utilized to quantify cerebrosides from patient specimens. Cerobrosides with a d18:1 backbone with fatty acids from 14 to 26 carbons were assessed. Molecular species of cerebrosides were quantified from pancreas tissues (**A**); and corresponding plasma samples (**B**) from patients with pancreatitis, non-metastatic (nodal negative) pancreatic cancer, or metastatic (nodal positive) pancreatic cancer. * *p <* 0.05 assessed by *t*-test analysis.

Sphingolipid analyses were extended to the cerebrosides (Figure 2). The cerebroside class of sphingolipids are glycosylated ceramides with a glucose or galactose molecule attached. Again, analyses included quantification of cerebroside molecular species in both pancreas tissues (Figure 2A) and corresponding plasma (Figure 2B). In regards to pancreatic tissue, while C16:0 cerebrosides demonstrated large variability in the groups with pancreatic cancer, cerebrosides consisting of a C18:0 (*p <* 0.05 either group), C20:0 (*p* < 0.05 for nodal negative, *p* < 0.005 for nodal positive), C22:0 (*p* < 0.05 for nodal negative, *p* < 0.005 for nodal positive), C24:0 (*p* < 0.01 for nodal negative, *p* < 0.05 for nodal positive), or C24:1 (*p* < 0.05 for either group) fatty acid demonstrated significant reductions regardless of metastasis status. In contrast to the tissue, plasma cerebroside species (C16:0, C20:0, C22:0, C24:0, C24:1) were significantly elevated from nodal positive pancreatic cancer patients, but not nodal negative patients (Figure 2B). 

**Figure 3:**
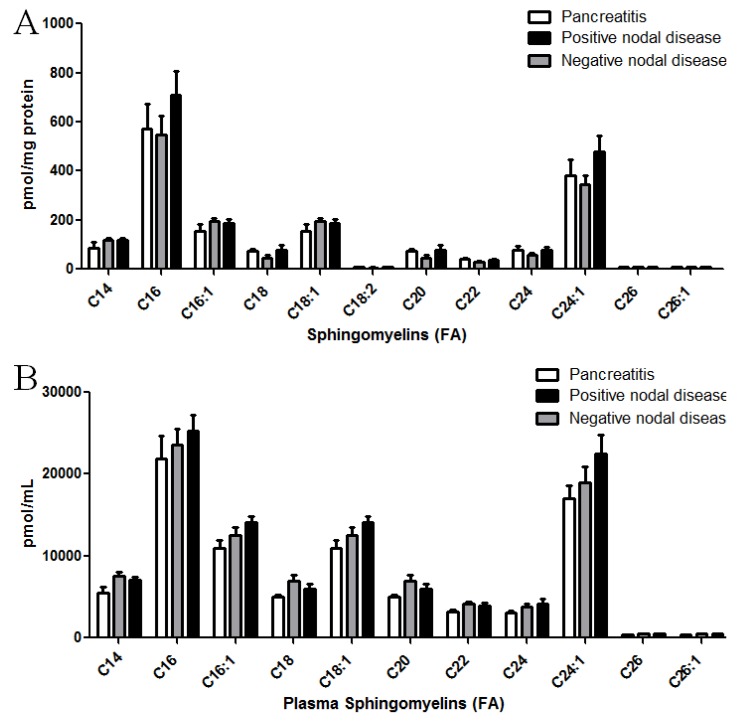
Sphingomyelin levels in patient specimens with pancreatic cancer. LC-MS/MS was utilized to quantify sphingomyelin levels from patient specimens. Sphingomyelins with a d18:1 backbone with fatty acids from 14 to 26 carbons were assessed. Molecular species of sphingomyelins were quantified from pancreas tissues (**A**) and corresponding plasma samples (**B**) from patients with pancreatitis, non-metastatic (nodal negative) pancreatic cancer, or metastatic (nodal positive) pancreatic cancer. * *p <* 0.05 assessed by *t*-test analysis.

Sphingomyelin species were also quantified from pancreatic cancer specimens (Figure 3A) and corresponding plasma (Figure 3B). Alterations in these choline-containing ceramide metabolites were not observed in the tissues or plasma from any of the patient populations. 

**Figure 4:**
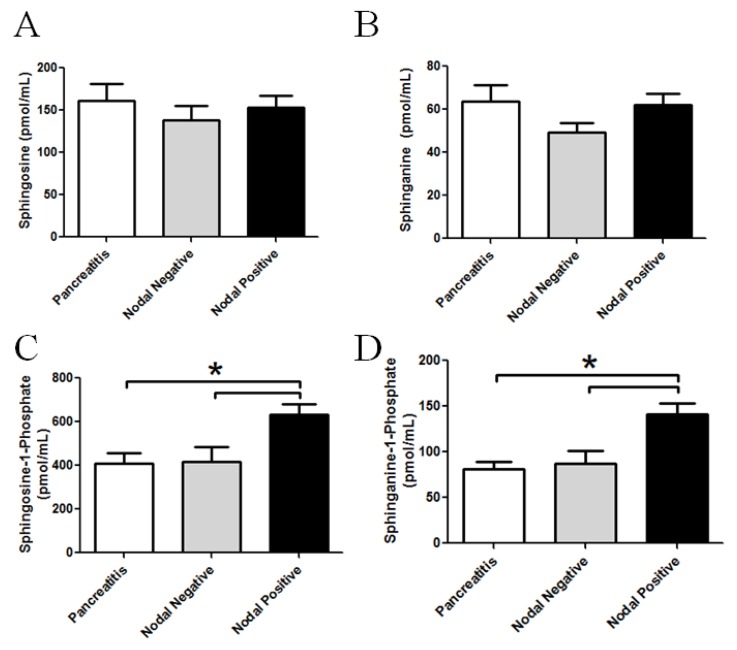
Altered plasma phosphorylated long chain sphingoid bases in patient samples with pancreatic cancer. LC-MS/MS was utilized to quantify perturbation in the long chain bases from plasma. Graphical representations of the data for (**A**) sphingosine; (**B**) sphinganine; (**C**) sphingosine-1-phosphate; and (**D**) sphinganine-1-phosphate are shown from the plasma from patients with pancreatitis, nodal negative pancreatic cancer or nodal positive pancreatic cancer. * *p <* 0.05 assessed by t-test analysis.

The long chain bases, sphingosine and sphinganine, as well as their phosphorylated metabolites, sphingosine-1-phosphate (S1P) and sphinganine-1-phosphate (dhS1P), were also quantified from plasma samples of patients, with or without nodal disease, as well as from patients with pancreatitis. (Figure 4). While no significant alterations in plasma sphingosine or sphinganine levels could be detected, S1P and dhS1P mass significantly increased in the plasma from nodal positive pancreatic cancer specimens. Specifically, S1P showed a significant increase in the plasma from patients with nodal disease compared to the pancreatitis group (*p =* 0.022) or the nodal negative group (*p =* 0.027). DhS1P showed the same significant increase in plasma from patients with nodal disease when compared to patients with pancreatitis (*p <* 0.01) or patients with non-metastatic pancreatic cancer (*p <* 0.02). 

We also assessed the mass of long chain sphingoid bases in pancreatic tissues (Table 1). No significant differences between pancreatitis, nodal negative and nodal positive disease for sphingosine, sphinganine, and sphingosine-1-phosphate were observed. Sphinganine-1-phosphate was below the limit of quantification in most tissues. Taken together, phosphorylated and glycosylated ceramide metabolites are elevated in the plasma from, but not the tissues of, pancreatic cancers (Figure 2, Figure 4, Table 1).

**Table 1:** Pancreatic Tissue Long-chain Bases (pmol/mg protein).

Lipid	Pancreatitis	Nodal Negative	Nodal Positive
Sphingosine	53.88 ± 27.55	43.96 ± 9.12	58.59 ± 10.29
Sphinganine	6.48 ± 2.95	8.03 ± 3.27	11.57 ± 1.73
Sphingosine-1-phosphate	2.15 ±1.72	1.19 ± 0.20	0.7918 ± 0.09

### 2.2. Deregulation of Growth Factors and Cytokines in Patients with Pancreatic Cancer

A panel of growth factors and cytokines that are related to or suggested to be related to, pancreatic tumorigenesis was analyzed by a luminex-based assay. Compared to non-cancerous pancreatitis plasma samples, the interleukins IL-6 and IL-8 were significantly elevated (*p <* 0.05) in nodal negative and nodal positive pancreatic cancer patients. (Table 2) CCL11 and IP-10 also showed significant elevation (*p <* 0.01 and *p <* 0.05 respectively) in patients with positive nodal, but not non-nodal disease compared to pancreatitis. (Table 2) In contrast, TNFα levels were increased in nodal negative, but not nodal positive disease, as compared to pancreatitis control samples. Several factors, including IFNγ, sFasL, and VEGF (*p <* 0.08), did not demonstrate any significant differences between groups. 

**Table 2:** Plasma Cytokine/Growth Factor levels.

Cytokine/Growth Factor	Pancreatitis	Nodal Negative	Nodal Positive
CCL11	0.538 ± 0.078	0.714 ± 0.084	0.795 ± 0.042 *
IFNγ	0.944 ± 0.145	1.399 ± 0.184	1.196 ± 0.092
IL-6	0.204 ± 0.034	0.392 ± 0.055 *	0.379 ± 0.049 *
IL-8	0.320 ± 0.045	0.545 ± 0.066 *	0.534 ± 0.047 *
sFasL	0.175 ± 0.026	0.226 ± 0.032	0.209 ± 0.017
VEGF	0.933 ± 0.188	1.42 ± 0.159	1.339 ± 0.105
TNFα	0.273 ± 0.044	0.470 ± 0.052 *	0.366 ± 0.032
IP-10	0.846 ± 0.091	1.228 ± 0.187	1.772 ± 0.252 *

* Statistically significant from pancreatitis assessed by *t*-test analysis.

**Figure 5:**
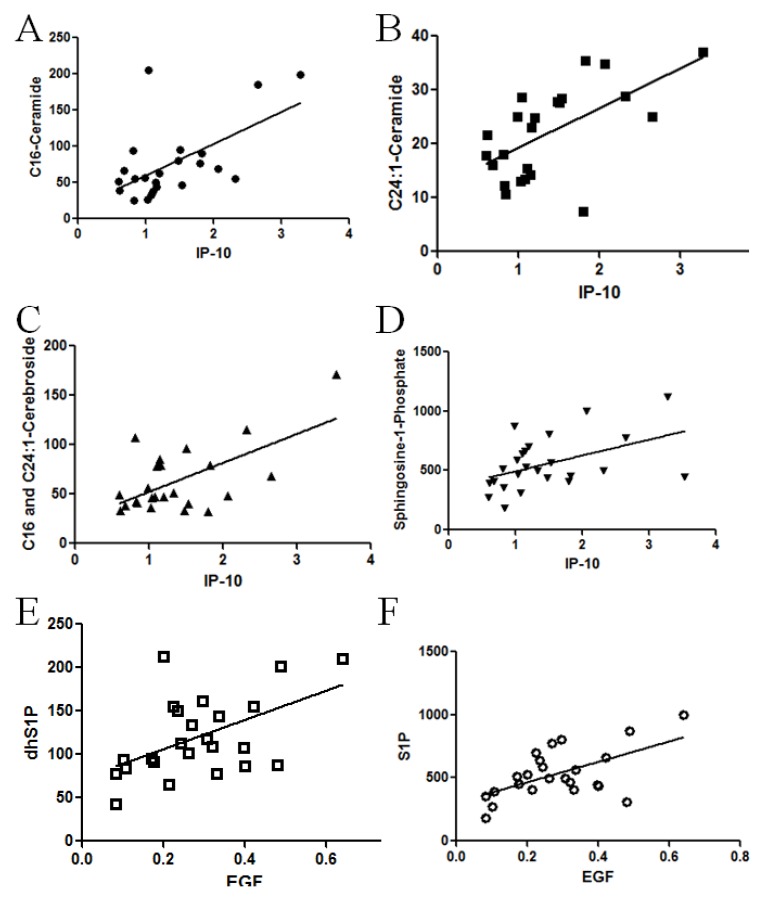
Correlation between cytokines and sphingolipid alterations in patient specimens with pancreatic cancer. To determine if a statistical relationship exists between cytokines and sphingolipids in patient specimens, Pearson correlation analysis was performed. (**A**) Tissue C16-ceramide and plasma IP-10 levels (*r =* 0.583, *p =* 0.0035); and (**B**) tissue C24:1-ceramide and plasma IP-10 levels from tissue demonstrate a positive correlation (*r =* 0.6012, *p =* 0.0024); (**C**) as did plasma C16:0 and C24:1 cerebroside mass (*r =* 0.6174, *p =* 0.0013), with plasma IP10; and (**D**) plasma S1P with IP10 (*r* = 0.4655, *p* = 0.019). Plasma EGF levels demonstrated positive correlated with both plasma; (**E**) S1P (*r =* 0.6928, *p =* 0.0001) and (**F**) dhS1P (*r =* 0.6787, *p =* 0.0002) levels.

Correlation analysis was next utilized to determine if a statistical relationship exists between these elevated plasma cytokines and altered sphingolipids in pancreatic patients. Pearson correlation analysis was applied to evaluate differences between the level of circulating growth factor or cytokines and the plasma or tissue level of ceramide, cerebrosides, S1P and dhS1P. The analysis revealed a positive relationship between sphingolipid metabolites and IP10 (Figure 5). Specifically C16 and C24:1-ceramide tissue mass demonstrated a positive correlation (Figure 5A,B, *r =* 0.583, *p =* 0.0035 and *r =*0.6012, *p =* 0.0024 ,respectively) as did C16:0 and C24:1 plasma cerebroside mass (Figure 5C. *r =* 0.4886, *p =* 0.0154 and *r* = 0.6787, *p =* 0.0003, respectively) with IP10. This correlation was also observed with circulating S1P (Figure 5D, *r* = 0.4655, *p* = 0.019) and dhS1P (*r* = 0.4499, *p* = 0.024 data not shown)). Despite a significant increase in plasma CCL11, IL-6, and IL-8 in pancreatic cancer patients, these proteins did not correlate with any of the lipid changes (data not shown). Though plasma EGF levels did not reach significance for pancreatic cancer patients, EGF levels positively correlated with both S1P (Figure 5E, *r =* 0.6928, *p =* 0.0001) and dhS1P (Figure 5F. *r =* 0.6787, *p =* 0.0002) levels. No correlation was observed between any of the lipid species and IFNγ, sFasL, TNFα, or VEGF (data not shown). Furthermore, no significant correlation was observed between decreased tissue cerebroside levels and any of the cytokines and growth factors assessed.

**Figure 6:**
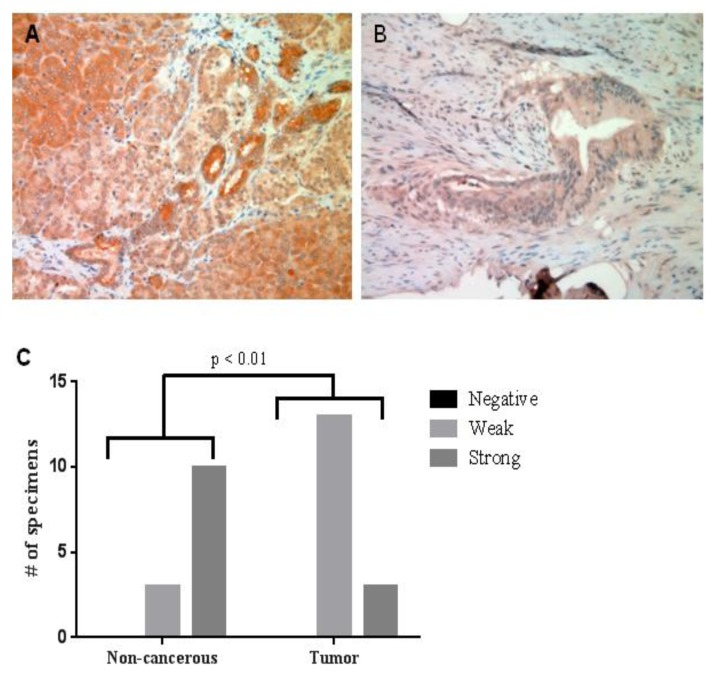
IP10 expression in pancreatic tumor specimens. IP 10 immunohistochemistry staining shows IP10 expression was down-regulated in tumor tissue (**B**) compared with non-cancerous, tissue (**A**)**.** (**C**) Fisher’s exact test shows the quantitative staining intensity difference between tumor and normal tissue is significant (*p* < 0.01).

Immunohistochemistry was used to determine if the elevated plasma levels of IP10 that correlated with sphingolipid changes in pancreatic cancer were due to elevated expression of IP10 in the pancreatic tumors (Figure 6). Interestingly, the expression of IP-10 was significantly down-regulated in cancer specimens (Figure 6B) compared to non-cancerous pancreatic tissue from patients who underwent resection for other reasons (Figure 6A). Even though tumor mass of ceramide species, as well as plasma mass of glycosylated and phosphorylated metabolites, positively correlated with elevated circulating IP10 levels in nodal positive pancreatic cancers, pancreatic tumors exhibited less IP10 staining intensity. Taken together, combinatorial lipidomic, Luminex and immunohistochemical analyses reveal correlated changes in specific tissue and plasma sphingolipid metabolites and cytokines in pancreatic cancer as a function of nodal disease.

## 3. Experimental Section

### 3.1. Pancreatic Cancer Clinical Specimen

After obtaining approval by the Institutional Review Board (IRB) of Penn State Hershey, 20 defined (stage, type, presence of regional metastasis) pancreatic cancer clinical specimens (ductal adenocarcinoma) as well as 5 non-cancerous pancreatitis clinical specimens were collected. Corresponding plasma samples from these 25 patients were also obtained. 

### 3.2. Lipid Extraction and Mass Spectrometry Analysis of Tumor Specimen and Plasma

Analyses focused on pancreatic tissue obtained from human patients with pancreatitis, a chronic inflammation of the pancreas that can lead to pancreatic cancer, and pancreatic tissue taken from patients with nodal and non-nodal pancreatic cancer. Lipid extracts from tissues were prepared as previously described (10) and the sphingolipid quantification was determined by LC-MS/MS utilizing an Agilent 1100 HPLC system coupled to an AB Sciex 4000 QTrap mass spectrometer [13]. Pancreas samples were homogenized by sonication in 1:10 diluted PBS. Protein assays were performed and typically 1 mg of protein was used for each sample preparation. For plasma samples, 30 μL of sample was utilized. 

### 3.3. Cytokine Analysis

To quantify cytokine and growth factor plasma levels, a Procarta Cytokine Assay Kit (Affymetrix, Santa Clara, CA, USA) was utilized according to manufacturer’s instructions. Based upon correlation analysis with sphingolipid metabolites, IP10 expression from pancreatic tumor specimens was quantified by immunohistochemical analysis. Specifically, 5 μm thick sections of formalin-fixed, paraffin-embedded tumor specimens were de-paraffinized in xylene and rehydrated. Antigen retrieval was performed with antigen retrieval buffer (Dako, Carpinteria, CA, USA) for 10 min at 95 °C. Endogenous peroxidase was blocked using 3% hydrogen peroxide in PBS for 12 min. The specimens were incubated for 20 min at room temperature with a protein-blocking solution consisting of PBS (pH 7.5) containing 5% normal horse serum and 1% normal goat serum and then incubated at 4 °C in a 1:200 dilution of human IP10 antibody overnight. The samples were then rinsed and incubated for 1 h at room temperature with peroxidase-conjugated anti-rabbit IgG. Next, the slides were rinsed with PBS and incubated for 5 min with diaminobenzidine (Research Genetics, Huntsville, AL, USA). The sections were washed three times with PBS, counterstained with Mayer’s hematoxylin (Biogenex Laboratories, San Ramon, CA, USA), and washed once each with distilled PBS and water. Afterward, the slides were mounted using Universal Mount (Biomeda, Foster City, CA, USA) and examined using a bright-field microscope. A positive reaction was indicated by a reddish-brown precipitate in the cell. Depending on the percentage of positive cells and staining intensity, IP10 staining was classified into three groups: negative, weak positive, and strong positive. Specifically, the percentage of positive cells was divided into five grades (percentage scores): <10% (0), 10–25% (1), 25–50% (2), 50–75 (3), and >75% (4). The intensity of staining was divided into four grades (intensity scores): no staining (0), light brown (1), brown (2), and dark brown (3). Sp1 staining positivity was determined by the formula: overall scores = percentage score × intensity score. The overall score of ≤3 was defined as negative, of >3–≤6 as weak positive, and of >6 as strong positive. Fisher’s exact test was used for comparison. 

Data analysis Correlation analysis, linear regression analysis, and *t*-test analysis were performed using GraphPad Prism 5.0 software, with statistical significance considered if *p <* 0.05. 

## 4. Conclusions

Although the biological activity of sphingolipids has been extensively explored in cell culture and animal models, it remains unclear what the exact “*in vivo*” roles of sphingolipids are in human cancers. Here, we first report a comprehensive sphingolipid analysis in human pancreatic clinical specimens. 

The data demonstrate that specific species of ceramide and cerebroside are significantly and selectively altered in the cancerous tissue comparing to the non-cancerous pancreatic tissue. Specifically, we observe significant elevation of C16 and C24:1 ceramides in nodal positive pancreatic tissue (Figure 1A). Increasing levels of ceramides in the cancer tissue is a surprising finding as ceramide is generally considered to be a “pro-apoptotic” lipid and thus it theoretically should be down-regulated in cancer cells. However, this conclusion is largely supported by studies conducted in *in vitro* cell culture models. In contrast, recent studies suggested that certain species of ceramide may be elevated in some human malignancies. For instance, C16:0, C24:0, and C24:1 ceramide are up-regulated in squamous cell carcinoma of the head and neck (SCCHN) [14,15]. SCCHN showed a contrasting decrease in C18-ceramide, however, the baseline C18:0 ceramide was quite low in pancreatic tissue and no significant differences were observed. Similar elevations of specific ceramides in breast cancers have also been observed [16]. It has been postulated that the accumulation of C16-ceramides may have proliferative properties, which are in opposition to the apoptotic/growth arresting properties of C18-ceramides [17]. Work by others have reported an apoptotic role of C16-ceramide and a proliferative role of C24-ceramides [18]. Taken together, the selective change of ceramide molecular species in cancer requires further investigation into their involvement in disease pathogenesis. In particular, the specific ceramide synthases that generate C16 and C24:1 ceramide species in pancreatic cancer as well as quantitative changes in corresponding acyl-CoAs need to be explored. 

A unique aspect of the present study is the elevation of glycosylated and phosphorylated ceramide metabolites in plasma samples from pancreatic cancer patients. While *in vitro* studies suggest that phosphorylated and glycosylated ceramide metabolites can be shed or exocytosed from tumors, it is too early to speculate if the reduced cerebroside content observed in pancreatic tumors directly correlates with the elevation of cerebrosides observed in the plasma from pancreatic cancer patients. However, the observation that cerebrosides are decreased in pancreatic tumors does offer some interesting speculation. In general, the biological effect of cerebrosides is thought to be related to drug resistance via upregulation of multidrug resistance gene (MDR1) in cancer cells [19,20]. Considering the drug resistance of pancreatic cancer, this result was unexpected. As we do not observe a direct dichotomy between ceramide and cerebroside changes in terms of fatty acyl substituents, it appears unlikely that cerebroside catabolism is responsible for elevated ceramides. Since cerebrosides are the first committed step towards higher order glycosphingolipids, which were not investigated in this study, further studies are needed to examine changes in sulfatides, gangliosides and neutral glycosphingolipids, such as globosides. This will allow determination if cerebrosides are potentially being metabolized into these higher order classes at a faster rate. Microarray analyses by others have revealed increased galactosylceramidase [21], increases in several fucosyltransferases (FUT3, 6, 8) [22,23,24], and sialytransferase 6 [31,32]. These alterations would suggest a shift away from sulfatide containing lipids (typically metabolized from galactosylceramide) and the formation of fucosylated and sialyated gangliosides. It should be noted that the circulating antigen (CA) 19-9 antigen, an often used biomarker for pancreatic cancer, can be found on gangliosides. In fact, these Sialyl Lewis(a) gangliosides correlate with circulating CA 19-9 levels [25]. 

Other significant lipid alterations observed were the elevation of the phosphorylated sphingolipids, S1P and dhS1P, which are selectively increased in patients with nodal positive pancreatic cancers. It has been recognized and accepted by most researchers that sphingosine kinase acts like an oncogene and is upregulated in many human cancers [26,27,28,29]. The elevation is associated with regional lymph node metastasis just as the tissue ceramides and plasma cerebrosides were. Mechanistically, S1P and dhS1P are typically considered pro-inflammatory, pro-mitogenic, and/or chemotaxic lipids that may serve to facilitate the progression of pancreatic cancer. This idea is further supported by recent work in the Ogretmen laboratory where it was demonstrated that systemic sphingosine kinase 1-generated S1P regulates metastatic potential, but not tumor S1P, via regulation of tumor S1PR2/Brms1 signaling [30]. These perturbations in phosphorylated ceramide metabolites also have the potential to be used as biomarkers to risk stratify patients. However, much care would be needed as elevated S1P and dhS1P are observed in other diseases, such as models of diabetes [31], a risk factor for pancreatic cancer. As another example, S1P has been demonstrated to be more predictive of obstructive coronary artery disease than other well-established risk factors, such as family history, diabetes, and hypertension [32]. However, coupling phosphorylated sphingosines/sphinganines with other biomarkers, such as CA 19-9, has the potential to increase the predictive power of these markers.

Mounting evidences suggested cross talks between growth factors and sphingolipid signaling pathways. Long *et al*. demonstrated that S1P-mediated ERK activation could be completely abolished by a Her2 inhibitor or antisense mRNA to Her2 in breast cancer cells [33]. Moreover, AG1478, an EGFR inhibitor, attenuated the activity of sphingosine kinase1 (SphK1) [34]. On the other hand, EGF can stimulate the activity of SphK1 in breast cancer cells [35]. Among the cytokines and growth factor assessed, several growth factors and cytokines including IL-6, IL-8, CCL11, and IP-10 are higher in patients with pancreatic cancer. However, only elevated IP10 showed significant positive correlations with some of the sphingolipid levels as shown in Figure 5. There is a complex interaction between the host and the tumor and it is not clear which factors are host-derived *versus* tumor-derived. We had expected to observe elevated IP-10 within the pancreas, due to the ability to recruit mast cells, which are increased in pancreatic cancer [36], and were subsequently surprised to observe reduced staining. Thus, it is likely that systemic IP-10 is not tumor-derived or reflects infiltrated immune cells, unless it is being secreted faster than it is being produced in pancreatic cancer tissue. It is still too early to speculate on the mechanisms by which tissue or plasma levels of sphingolipids contribute to elevated circulating cytokines or growth factors or how circulating growth factors or cytokines regulate sphingolipid metabolism in pancreatic cancers. Confounding some of this interpretation is the comparison to pancreatitis sample, which is an inflammatory disease itself. However, establishing the ability of these several proteins and lipids to distinguish between pancreatic cancer and pancreatitis as well as between pancreatic cancers with distinct metastatic potential is an important step in the understanding of pancreatic cancer. The exact link between these lipids and proteins remain unknown and will require further study. 

Lipidomics is a technology analyzing a large spectrum of lipid species to facilitate characterizing the biological roles of each lipid. Here, we utilized a mass spectrometry-based sphingolipid analysis in human pancreatic cancer clinical specimens. We demonstrate aberrant sphingolipid metabolism in the tissues and plasma of patients with pancreatic cancer. The deregulation of sphingolipid metabolism is associated with more advanced and aggressive disease that could potentially impact the long-term survival of pancreatic cancer patients. Therefore, like genomics and transcriptomics, metabolomics strategies such as lipidomics could be used in risk stratify patients and provide further understanding of pancreatic cancer development and progression.
